# Supplementary material for: Do Children with Attention-Deficit/Hyperactivity Disorder Follow a Different Dietary Pattern than That of Their Control Peers?
Source: Nutrients. 2022 Mar 8;14(6):1131. doi: 10.3390/nu14061131 (PMC8949924; doi:10.3390/nu14061131)
Supplement: Supplementary file 1 [file nutrients-14-01131-s001.zip › nutrients-1611594-supplementary.pdf]

Table S1. Food consumption in grams/day by age group and ADHD diagnosis

	Preschool-age children				Elementary school-age children			
	ADHD n = 57	CONTROL n = 202	*p* Raw	p^1 Adjusted	ADHD n = 213	CONTROL n = 262	*p* Raw	p^1 Adjusted
**Starch**	166.98 (62.90)	155.89 (64.79)	0.252	0.139	189.06 (71.14)	186.99 (68.48)	0.747	0.672
Savory cereals & tubers	120.49 (54.69)	111.67 (50.89)	0.257	0.157	143.97 (62.51)	143.14 (61.68)	0.885	0.594
Sweet cereals	46.49 (28.37)	44.22 (29.89)	0.609	0.454	45.09 (27.94)	43.85 (28.79)	0.635	0.901
**Legumes**	9.59 (7.60)	9.09 (5.18)	0.564	0.695	13.47 (7.90)	12.22 (8.47)	0.098	0.168
**Nuts**	2.96 (4.37)	2.55 (3.24)	0.437	0.463	3.01 (4.01)	2.75 (3.57)	0.466	0.497
**Vegetables**	94.44 (51.09)	94.52 (52.04)	0.992	0.964	99.01 (53.93)	100.61 (59.51)	0.760	0.854
Cooked vegetables	69.87 (36.85)	67.11 (38.34)	0.629	0.559	73.59 (41.99)	73.42 (43.63)	0.965	0.617
Raw vegetables	24.57 (26.32)	27.40 (28.97)	0.506	0.388	25.42 (25.32)	27.20 (27.47)	0.467	0.228
**Fruit**	389.88 (195.07)	352.04 (169.04)	0.151	0.431	337.60 (167.76)	334.50 (167.66)	0.841	0.079
Fresh fruit	99.94 (79.73)	110.23 (59.99)	0.290	0.357	93.35(64.91)	104.60 (67.22)	0.066	0.115
Preserved fruit	8.72 (17.03)	3.43 (9.12)	**0.002**	**0.007**	5.01 (12.56)	5.13 (14.61)	0.928	0.284
Fresh fruit juice	48.54 (73.23)	41.64 (56.36)	0.447	0.560	38.74 (56.42)	47.35 (65.24)	0.129	0.060
Commercial fruit juice	90.95 (86.10)	78.18 (89.27)	0.337	0.940	61.18 (70.12)	55.63 (68.24)	0.384	0.673
**Dairy products**	416.53 (156.77)	421.22 (162.00)	0.846	0.241	392.17 (157.53)	391.89 (157.60)	0.984	0.825
Milk	261.64 (145.34)	168.59 (138.31)	0.741	0.106	239.98 (130.15)	244.27 (130.00)	0.721	0.670
Cheese and yogurt	98.34 (59.54)	105.88 (62.15)	0.416	0.735	105.24 (63.05)	96.59 (61.57)	0.133	0.071
Dairy desserts	56.55 (47.12)	46.76 (42.23)	0.133	0.177	46.96 (41.96)	51.03 (42.21)	0.295	0.609
**Olive oil**	32.19 (7.53)	29.98 (8.16)	0.067	0.063	10.04 (4.79)	10.30 (3.75	0.513	0.139
**Protein foods**	154.37 (63.73)	157.93 (59.98)	0.696	0.980	173.49 (63.20)	169.52 (59.90)	0.483	0.276
Meats	92.06 (37.76)	94.03 (40.26)	0.741	0.932	110.61 (43.67)	107.24 (43.52)	0.402	0.161
White meat	31.62 (16.32)	31.34 (16.23)	0.908	0.690	39.23 (19.72)	37.52 (19.48)	0.344	0.102
Red and processed	60.44 (28.17)	62.69 (30.30)	0.615	0.916	71.38 (31.39)	69.71 (32.29)	0.572	0.362
Fish	45.14 (29.01)	46.55 (28.73)	0.743	0.766	45.52 (31.28)	45.43 (29.88)	0.975	0.935
Oily fish	14.61 (14.99)	18.58 (18.00)	0.130	0.091	17.13 (17.71)	18.65 (17.73)	0.353	0.925
White fish	29.43 (24.14)	30.96 (21.31)	0.643	0.851	23.46 (18.43)	24.25 (17.67)	0.634	0.984
Seafood	2.06 (2.25)	2.37 (3.17)	0.491	0.767	3.10 (4.16)	3.26 (4.00)	0.657	0.780
Eggs	17.17 (11.91)	17.35 (10.21)	0.911	0.737	17.36 (8.65)	16.85 (9.68)	0.550	0.375
**Sweets**	11.64 (9.31)	11.28 (8.62)	0.788	0.579	11.28 (10.73)	11.84 (9.66)	0.552	0.195
**Sodas**	19.97 (43.02)	21.22 (44.18)	0.850	0.902	38.45 (58.71)	37.42 (57.70)	0.874	0.898

ADHD Attention deficit hyperactivity disorder; CONTROL Children without ADHD. Mean (SD). P^1 Adjusted value by sex, socioeconomic level, phamacological treatment, IQ, internalizing problems (CBCL) and ASD comorbidity. Significant differences in bold p <0.05.

Table S2. Spanish Diet Quality Index scores

	Preschool-age children				Elementary school-age children			
	ADHD n = 42	SUBCLINICAL n = 31	CONTROL n = 186	p	ADHD n = 168	SUBCLINICAL n =4 5	CONTROL n = 262	p
SDQI*	63.60 (7.37)	61.67 (6.69)	62.89 (7.31)	0.668	60.85 (7.71)	59.53 (6.96)	60.18 (7.91)	0.517
Unhealthy	4.80% (2)		3.50% (7)	0.905	7.7% (13)	8.90% (4)	7.60% (20)	0.992
Need to improve	95.2% (40)	100% (15)	96.00% (194)		92.3% (155)	91.10% (41)	92.40% (242)	
Healthy			0.5% (1)					

SDQI Spanish Diet Quality Index scores

Mean (SD); Percentage (n)
